# A qualitative study on the implementation of family health team: the perspectives of providers and patients

**DOI:** 10.1186/s12875-020-01217-7

**Published:** 2020-08-09

**Authors:** Ainul Nadziha Mohd Hanafiah, Mohammad Zabri Johari, Syafinas Azam

**Affiliations:** 1 Institute for Health Systems Research, National Institutes of Health, Ministry of Health Malaysia, Shah Alam, Selangor Malaysia; 2 Institute for Health Behavioural Research, National Institutes of Health, Ministry of Health Malaysia, Shah Alam, Selangor Malaysia

**Keywords:** Primary healthcare, Family health team, EnPHC

## Abstract

**Background:**

Malaysia has committed to the global call to achieve universal health coverage, and with the adoption of Sustainable Development Goals, is further strengthening the health system through the primary health care services, particularly the family doctor concept. The Enhanced Primary Health Care (EnPHC) initiative was implemented to address the worrying upward trend of non-communicable disease prevalence, and incorporates the Family Health Team (FHT) concept. The aim of this paper is to describe the implementation of the FHT as part of the EnPHC intervention.

**Methods:**

In-depth interviews and focus group discussions were conducted with the intervention design team, healthcare providers and patients in two rounds during the implementation period. A total of 121 individuals in the two rounds, split into different groups, where some of the participants of the FGD were also interviewed individually. Data were analysed using a thematic analysis, with codes being organised into larger themes.

**Results:**

Themes that emerged from the data were around the process of FHT implementation and the advantages of the FHT, which included continuity of health care and improved quality of care. Patients and health care providers were receptive to the FHT concept, and took the effort to adapt the concept in the local settings.

**Conclusions:**

The FHT concept implemented at 20 public primary health clinics has benefits appreciated by health care providers and patients. Addressing the viable shortcomings would better prepare the current primary healthcare system to scale up the FHT concept nationwide and enhance its feasibility and sustainability.

**Trial registration:**

The study is registered with the National Medical Research Register, Ministry of Health Malaysia (NMRR-17-295-34711).

## Background

Universal health coverage (UHC) is a key component of the Sustainable Development Goals (SDG) and has been the goal of most countries globally since the Alma Ata Declaration in 1978 [1]. Goal 3 of the SDG is about “ensuring healthy lives and promoting the well-being for all at all ages”, and specifically to “achieve universal health coverage, including financial risk protection, access to quality essential healthcare services and access to safe, effective, quality and affordable essential medicines and vaccines for all” [2]. When adopting the SDG in 2015, Governments made the pledge to enhance the wellbeing of citizens by achieving UHC, where the population is ensured access to, and receiving needed good quality health services in the form of health promotion, preventive care, treatment, rehabilitation, and palliative care, without facing financial adversity [1]. The primary health care (PHC) system has been under the spotlight to ensure that UHC and betterment of population health are achieved [3].

Malaysia, in her pledge towards UHC, emphasised on efforts to improve the existing PHC system by strengthening the family medicine services in the public sector. The ‘womb-to-tomb’ scope of services encompasses aspects of the entire life-course, from pregnancy to elderly care. The Family Doctor Concept (FDC) was initiated in response to the need to further strengthen the PHC services for universal health coverage through achieving ‘one family one doctor’ and personalised care [4]. The FDC has been adopted by countries worldwide with the common goal of providing first point of contact, comprehensive, patient-centred healthcare services to patients and their families [5, 6]. In Canada, the teams are multidisciplinary, offering enhanced access to team-based care to the general population [7]. There was improved access to healthcare services, greater internal coordination, broader scope of services provision, as well as enhanced patient-centredness and patient support [8] and showed the most improvements in diabetes care compared to other primary care models in Canada [9]. However, in Hong Kong, health seeking behaviour did not fulfil the family doctor concept despite public understanding on the concept [5]. In Malaysia, the FDC was introduced at the end of 2014 in phases [10]. It has since been implemented in over 213 clinics in Malaysia [personal communication between ANMH with FDC desk officer at Family Health Development Division]. The FDC is a team comprising of healthcare providers from different disciplines that provides personalised and holistic care to patients, with greater reach of the population. Teams, comprising of doctors, nurses, pharmacists and assistant medical officers (AMO), with additional categories where possible, are responsible for populations in designated zones. The clinic coverage area is essentially divided into 2 or more zones with equal population size. The number of zones corresponds to the number of teams at the clinics. In Malaysia, AMOs are medical assistants or physician assistants who are not doctors.

Efforts are warranted to address the current worrying issue of the alarming rise in non-communicable diseases (NCD), which has changed the disease landscape in Malaysia [11]. The prevalence of diabetes mellitus, hypertension and hypercholesterolaemia have been showing an increasing trend [12, 13]. The Enhanced Primary Health Care (EnPHC) intervention was developed and initiated to introduce a systematic approach in managing NCD at primary care level through prevention, early detection and treatment [14] specifically to tackle issues in the comprehensiveness and continuity of care for NCD patients at primary care level through improvements in work processes at health clinics as well as hospital referral networks. The EnPHC intervention consists of a list of interventions, one of which is the Family Health Team (FHT), and is piloted at 20 PHC clinics in 2 states, Selangor and Johor. Selangor is more urbanised, with a larger population, while Johor has an equal urban-rural distribution. Both states make up 31.4% of the total Malaysian population of 32.58 million residents.

The Malaysian public healthcare service is heavily subsidised through general taxation. Patients only need to pay a nominal amount for registration and most treatments and procedures are covered by the subsidy (Safurah J, Kamaliah MN, Khairiyah AM, Nour HO, Healy J: Malaysia Health System Review, unpublished).

The FHT concept is a rebranding of the FDC specifically for the EnPHC intervention pilot, with no modifications in its concept or operationalisation. At clinics where the concept is new, its execution entailed several steps prior to its implementation. This included zoning of the clinic operational areas, formation of the multidisciplinary family health teams, and registration and assigning of the patients to specific teams based on their home addresses. Additionally, the public had to be made aware of the FHT model and briefed on the concept and its importance.

After approximately 10 months into the implementation of EnPHC initiative, how well was the FHT component received by the HCP and patients? And how well was the FHT component implemented in the views of HCP and patients?

This paper aims to describe the implementation of the FHT as part of the EnPHC intervention. A process evaluation of the EnPHC specifically of the FHT concept, was conducted by exploring the perceptions of healthcare providers (HCP) and patients towards the FHT implementation. The study is important to ensure the pilot project of EnPHC can be scaled up at a national level after taking into consideration existing facilitators and barriers.

## Methods

### Study design

This was a qualitative study which explored the experiences of the FHT members during implementation of the EnPHC intervention, as well as the experiences of patients 10 months into the intervention at the 20 clinics. This is to ensure that any issues during the intervention period could be explored, understood, and resolved before the scale-up of the intervention in the future.

### Settings

The study was conducted at 20 intervention clinics (9 in Selangor, 11 in Johor) in both rural and urban areas. The 20 clinics were matched based on location (urban or rural), number of daily clinic attendance, and number of medical doctors and Family Medicine Specialists. However, the physical (layout, electronic/ manual information system) and administrative set-up differ for every clinic. This provided a variation in feedback given by HCP and patients.

### Participants

Purposive sampling was used for this study. The unit of analysis were the health care providers (HCP) and patients who went to the clinics for services. The FHT sample population were the HCPs who were involved in the FHT services, while the patients were Malaysians who registered and attended regular follow-up appointments for at least 2 years at the same clinic, and attended at least 3 clinic (NCD) appointments during the EnPHC implementation period, to ensure that patients had at least 9 months of exposure to the intervention, regardless of the number of appointments during the period. Patients were recruited for interviews by the clinic Care Coordinators (typically nurse supervisors at the clinics) who identified patients who were most suited to provide relevant information for this study purpose, such as ability to converse in English or Malay. We also included members of the intervention design team to explore their intentions in the design and implementation of the EnPHC intervention. The sample size of patients was determined by data saturation.

### Data collection & tools

Data for this paper were derived from in-depth interviews (IDI) with clinic patients and focus group discussions (FGD) with the FHT and the intervention design team members, who are mid-level managers within the Ministry of Health (MOH).

**Fig. 1:**
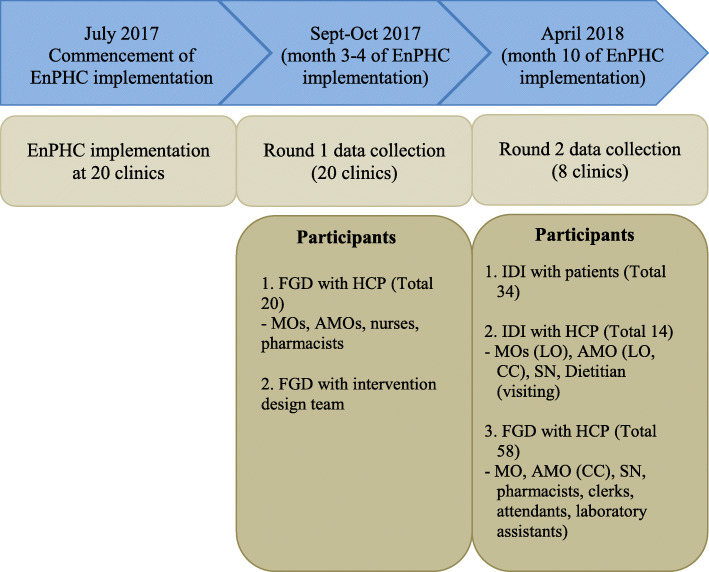
Schematic diagram on data collection. MOIC = medical officer in-charge; LO = liaison officer; MO = medical officer; CC = Care Coordinator; AMO = assistant medical officer; SN = staff nurse

Data collection was conducted in two rounds of interviews (Fig. 1). All 20 clinics were involved in the first round of interviews, about 2 months into the EnPHC implementation (September–November 2017). Only 8 clinics (4 in Selangor and 4 in Johor) were selected for the second round of interviews, approximately 10 months into the intervention implementation (April 2018). The FGD session with the intervention design team was conducted in the first round of interviews, patient interviews in the second round interviews, while FGDs with FHT were done in both rounds. Research team members [15] trained in qualitative research conducted all interviews and FGDs with FHT and patients face-to-face at the clinics, and with the intervention design team at their work place.

For the FGD with the clinic FHTs, a set of semi-structured interview questions was used to guide the interview. In the first round of data collection the FGD interviews focused on the process and feasibility of intervention implementation as well as the challenges faced. In the second round, the focus was on the acceptance, adoption, feasibility (i.e. barriers and facilitators), as well as perception on sustainability of the FHT. The FGD questions for the intervention design team centred mainly on the design, characteristics, execution plans and engagement mechanism of the FHT intervention development. Other areas covered were related to monitoring activities and management of issues encountered by the clinics. For the patient in-depth interview, the interview guide was developed based on several psychological and behavioural theories [16, 17]. The questions focused on their understanding of the clinics services and their awareness and perception on the FHT concept and its implementation.

Participant information sheets and informed consent forms were given to all participants prior to the interviews, which was preceded by a brief introduction of the interviewers and explanation of the study. Each IDI lasted between 30 to 120 min while FGD lasted between 60 to 200 min, with field notes taken by a note-taker. Interviews were audio-recorded and transcribed verbatim. Confidentiality was ensured where participants’ identifiers were removed from the transcripts. The research team cross-checked each transcript using audio-recordings and field notes.

### Analysis

Data were analysed using a thematic analysis. Transcribed interview verbatim were independently read by research team members to identify preliminary codes. An interpretivism approach was taken to interpret and code participants’ experiences in line with the details in the EnPHC standard operating procedure [14]. This way, coding of participants’ feedback was done without assumptions or subjective interpretation by the researchers. Meaning units were reviewed, identified, and sorted into codes before grouping them into categories. Lastly, through consensus, contents of each category group were summarised and grouped into main themes. The best representative participants’ quotes for each theme were chosen to support the results. Thematic analysis was used where initial open codes were generated from the data, after which the codes were organised into larger themes [18].

### Positionality of research team members

Research team members are researchers in the public research institutions, comprising of various ethnicities. Team members have been involved in researches in health systems and health behaviour. Team members were not involved in the development of the EnPHC intervention, but are users of the public healthcare services. All team members have been trained in conducting qualitative research and were involved in this study from the start until the end as neutral parties in assessing the feedbacks of participants of the interviews and FGDs without making assumptions on behalf of the study stakeholders or participants.

## Results

In the first round, a total of 20 members of the FHT consisting of doctors, AMOs, nurses and pharmacists between the ages of 30 and 47 years old, and eight individuals from the intervention design team (i.e. programme managers: individuals and institutions that participated in designing the EnPHC initiative) aged between 39 and 57 years old were interviewed. In round two, the participants comprised of 35 patients aged 25 to 76 years old (Table 1), and a total of 49 doctors, AMOs, nurses, pharmacists, dietician, as well as 10 others (clerks, attendants, lab assistants), from the FHT teams, all aged 25 to 57 years old. Details of the clinics are in Table 2.

**Table 1:** Characteristics of patient

Characteristics	Number of Informants
**Age groups**	
Below 40	1
40–49	8
50–59	8
60–69	11
70–79	6
**Gender**	
Male	15
Female	20
**Ethnicity**	
Malay	32
Chinese	1
Indian	1
Iban	1
**Education background**	
Academic degree	4
Secondary high school	17
Primary school	14
**Job**	
Housewife	4
Retiree	12
Employed	10
Unemployed	9
**Appointment frequency since July 2017**	
3	14
4	12
5	4
6	4
7	1

Note: All participants are Diabetic and/or Hypertension patients

**Table 2:** Features of EnPHC intervention clinics

Health Clinic	Family Health Team	Medical Officer	Assistant Medical Officer	Nurse	Community Nurse	Other health personnel	
						Visiting	Permanent
1	2	3	2	7	9	Family Medicine Specialist, nutritionist, physiotherapist, occupational therapist, dietitian	
2	4	12	non-zone	13	5	nutritionist, physiotherapist, occupational therapist, dietitian	
3	2	7	4	9	7	Family Medicine Specialist, nutritionist, dietitian	Physiotherapist, occupational therapist
4	2	4	2	3	1	Family Medicine Specialist, nutritionist, physiotherapist, occupational therapist, dietitian	
5	3	8	3	11	10	Nutritionist, dietitian	Family Medicine Specialist
6	2	8	2	4	non-zone	Family Medicine Specialist, nutritionist, physiotherapist, occupational therapist, dietitian	
7	2	4	3	3	0	Family Medicine Specialist, nutritionist, physiotherapist, occupational therapist, dietitian	
8	2	5	2	non-zone	non-zone	Nutritionist, physiotherapist, occupational therapist, dietitian	Family Medicine Specialist
9	3	6	3	5	3	Family Medicine Specialist, nutritionist, physiotherapist	
10	2	5	3	7	8	Physiotherapist, occupational therapist, dietitian	Nutritionist
11	2	3	3	1	2	Family Medicine Specialist, nutritionist, physiotherapist, occupational therapist, dietitian	
12	2	2	3	2	2	Family Medicine Specialist, nutritionist, physiotherapist, occupational therapist, dietitian	
13	2	3	4	13	7	Family Medicine Specialist, nutritionist, physiotherapist, occupational therapist, dietitian	
14	2	4	3	6	11	Family Medicine Specialist, nutritionist, physiotherapist, occupational therapist, dietitian	
15	2	4	2	4	0	Family Medicine Specialist, nutritionist, physiotherapist, dietitian	Occupational therapist
16	2	4	4	10	14	Family Medicine Specialist, dietitian	
17	2	6	7	6	1	dietitian	Nutritionist, physiotherapist, occupational therapist
18	2	12	4	6	9	dietitian	Family Medicine Specialist, nutritionist, physiotherapist
19	2	3	3	3	0	Family Medicine Specialist, physiotherapist, dietitian	
20	2	2	3	9	4	Nutritionist, physiotherapist, dietitian	

Participants shared their experiences and perceptions on the FHT implementation, its benefits, shortcomings, and challenges. In general, three themes were identified namely, the implementation of FHT concept, continuity of health care, and quality of health care.

Feedback given by participants were consolidated from both rounds, which encapsulates the similarities in their perception on the FHT implementation. And the findings do not differ between the two rounds.

Generally, there were positive sentiments across the board with respect to the implementation of the FHT concept at the clinics. Challenges in terms of teething issues were inevitable, however, HCP and patients acknowledged its benefits and initiated efforts to adapt to their local contexts.

### FHT implementation

Given that some clinics had already implemented the FDC concept prior to the EnPHC initiative, the FHT concept that was introduced as part of the EnPHC intervention was nothing new to these clinics, as it required no abrupt process adjustments. Where FHT was a new concept, patients had noticed subtle differences in service changes even though they were unable to identify precisely the new FHT service. Although they were unable to describe specifically the changes in services from the FHT initiative implementation, they noticed subtle differences and minor changes from what was present prior to the intervention. This was especially so at clinics where FHT was a new concept. At clinics with pre-existing FDC, FHT was not unfamiliar.

*“The difference that I noticed, which I didn’t notice in the past, is the ‘one doctor for one family’. It’s good because like today, I got an appointment and my wife registered to see the same doctor too” (Patient, Round 2)*

Execution of the FHT concept was preceded by the process of population zoning of the clinic operation area. At the clinics, it was found that the FHT concept facilitates management of patient flow and fair distribution of workload to the teams. Some teams applied a colour coding system for zones to ease identification of patients’ records and files. From the HCP perspective, the FHT concept enables mapping of patient attendance in the clinic operation areas, which assists in better planning of resources. One HCP shared on the process of zoning:

*“We looked at the percentage of attendance based on the zones. We studied this [patient attendance by zone] for 2 months and then we divide into teams. In May, we had TOT [training of trainers] by KKM [Ministry of Health]; from there [the TOT session] we planned in more detail for the Enhanced primary health care” (HCP, Round 1)*

Another crucial step in FHT execution is the setting up of multidisciplinary teams. One of the key members of the team is the Care Coordinator (CC) who is tasked to ensure coordination in the continuum of care delivered to patients [14]. Individuals appointed to carry out the CC role were those with supervisory responsibilities at the clinics. Typically, there are at least two CC in a clinic, corresponding to the number of zones. Other team members typically include doctors, pharmacists, nurses, and assistant medical officers.

It was noted that the execution of FHT concept varied from one clinic to another. The variation can be explained by differences in manpower strength and working space and layout such as consultation rooms at the clinics. These were found to be important elements in ensuring effective implementation of the FHT concept and providing health care efficiently.

It was unanimous that the main overarching issue in the FHT implementation was manpower shortage. Some clinics were provided with additional manpower for the purpose of FHT implementation, whilst others were not as lucky. Some clinics underwent staff reshuffling in order to fulfil the team configuration. Clinic staff voiced their concerns on how clinic functions were affected by the shortage, and some clinics had to settle for fewer zones than what they should have.

Patients shared the same sentiments on manpower shortage and voiced their concerns on the well-being of the clinic staff.

*“[We have] Two teams because that is the minimum [number of teams] that should be formed for FDC. We can’t form three [teams] due to lack of staff. Our clinic space is another issue, that’s why we end up with only two teams” (HCP, Round 1)*

*“I requested for them to add manpower, because I feel sorry for them [clinic staff]. The workload per se, me myself feeling stress when looking at their work burden” (Patient, Round 2)*.

The other chief complaint amongst HCP and patients was space constraints. Space issues did not allow as many FHTs in the clinics as was desired for patient consultation and related activities. Therefore, this required reconfiguration of the zoning system in some clinics, principally to reduce the number of zones. Despite population size and geographical layout which warranted more zones, one clinic found it necessary to reduce the number of zones to accommodate all teams in the clinic.

Teamwork was not only apparent within teams, but also between teams at the clinics, as evidenced in our findings. A few clinics employed a system where patients from different zones were seen on different days of the week. This allowed the clinics to implement a back-up system where one zone team attended to the patients while the other team helped with administrative and preparatory work for the consultation day, as well as helped ease patient load on busy days.

### Continuity of care

In general, HCP and patients concurred that the concept of FHT ensures continuity of healthcare delivered to patients. Patients expressed contentment in seeing the same doctor as the familiarity assures better monitoring of their health status. Familiarity with the FHT members also improved interaction between HCP and patients. With the establishment of rapport between them, patients seemed more willing to share information where health care is concerned, and adherence to clinic appointments were more guaranteed. The HCPs were of the same views that patients were gratified with the trust and rapport built with them. They perceived that patients were more open to disclose information which they were previously not willing to share when they were seeing different HCPs at every appointment before the implementation of FHT. In return, HCPs used the opportunity to familiarise themselves with the patients and their families, and improve management of care provided to them. Personalised care can be optimised for the patients and their families, which was one of the main aims of the EnPHC intervention.

*“I’m more comfortable with the nurses because we’re familiar with them, so I feel happier talking, like friends. They are friendly, so I’m not shy to tell them things.” (Patient, Round 2)*

*“Sometimes, patients don’t like it if the doctors keep changing. There are things that they will not tell. When they only see one [same] doctor, they are more open [to share]. When the doctors keep changing, the patients don’t want to repeat themselves. So, when we’re already familiar with the patients, we know them already, because not all of the patient’s history is written in there [patients’ medical records]” (HCP, Round 2)*

We learned that some patients were not contented with their respective zones because the team members they were assigned to were not their preferred teams. This was a concern to the HCPs as it could discourage patients from adhering to clinic appointments and jeopardise continuity of care.

*“Patients have said to me “I don't like the zone, doctor”. I explained that they need to adhere to the zoning based on their home addresses. The reason for not favouring the zone is because of the doctor who is a bit strict. So they want to be in the zone with the doctor they like” (HCP, Round 2)*

Another challenge faced by the clinics was high turnover of doctors, which has an impact on continuity of care.

*“Our staff here change frequently. Since we started with FDC in 2015, at first the Sister retired, then we had a Matron but left [after a short while]. Then we had no Matron for a long time. After some time we had a Sister who then moved away. It was only last year that we really had a Sister. It’s difficult [with these changes] because it makes it difficult to pass over [the task].” (HCP, Round 1)*

### Quality of care

The FHT implementation has in many ways enhanced clinic workflow through its organised system of zoning. Patients were seen by the HCPs in an orderly manner according to zones. This initiative, which seemed rudimentary to some, had managed to improve quality of care by aiding smoother patient movement within clinic. In addition, with FHT implementation and its multidisciplinary team approach, clinics without Family Medicine Specialists (FMS) are receiving regular scheduled FMS visits. Another benefit of the initiative is the enhanced competency of HCPs. This stems from the involvement of all team members in providing care for patients in their zones, demanding for the need to be competent in managing NCD patients.

*“Now since Enhanced (EnPHC), every paramedic, everyone (team members) is involved. Before this, only one person in charge of NCD, so only that person knows inside out and everything [about NCD]. So now everyone has to know [having competency in NCD] because everyone is in charge of their zone” (HCP, Round 2).*

Quality of care is also improved through the personalised care approach, which includes appointment reminder calls from clinic staff. This was an initiative by most clinics in their efforts to improve adherence to clinic appointments. Many patients applauded the effort and were appreciative of this.

*“Now, nurse will call. ‘Tomorrow at 10 [am] you have appointment with doctor’, she will remind us. It’s helpful, because like yesterday I thought I was supposed to take blood. But the nurse insisted that it was to see the doctor. When I got home from work I checked my [appointment] card and yes, I have to see doctor today” (Patient, Round 2)*

One of the chief complaints at the clinics is space constraint, which has resulted in sharing of rooms in most clinics. As a consequence, patient privacy is compromised and clinical examination proved to be challenging, compromising quality of care provided to patients, as shared by one HCP:

*“Another issue is rooms… that’s where fundus [fundoscopy] is done, and ECG too. The space is very small for MO [medical officer]. All in one place. And there’s no privacy for the patients. When they do fundoscopy they need to turn off the lights… doctor needs to examine patients, we need to do ECG…. All three at the same time”. (HCP, Round 2)*

Despite the many challenges faced at the clinics, HCPs were confident on the sustainability of the FHT concept and considered it as an important component of the EnPHC intervention. This is made easier by the zoning system that is already in place in most, if not all clinics. The zoning system was an initiative which originated from the maternal and child health (MCH) services.

The FHT concept is highly regarded by the HCPs as it ensures continuous, comprehensive, person-centred care for patients. As shared by the intervention design team, FHT is designed with the aim to provide personalised care by a team of healthcare providers rather than just one individual. Leveraging on the strong features of the FHT concept, it would ultimately improve quality of care. This would stem from the greater HCP-patient relationship, resulting in improved sense of belonging of the patients to his/her own healthcare matters.

*“When we are doing FDC concept now, we are seeing the same patient. So, usually what I’ll do is that, not only will the patient be seeing HCP in my zone, but I’ll see the patient myself. So, I’ll write the in next appointment to come back and see me. So, continuation of care, automatically we will know, because we’ll be seeing the same patient for the past I think like nearly 1 year” (HCP, Round 2)*

*“[Before EnPHC] one nurse has to take care of 300 patients, so how are they [patients] going to be personalised? So it should be that team [FHT]. In EnPHC, team will work together for personalised care” (Intervention design team, Round 1)*

## Discussion

The principal objective of the FDC when it was initiated in 2014 was to ensure continuity of care for patients and family [10], which differs elsewhere where the priority is to enhance access to primary healthcare [7]. The implementation of the EnPHC intervention saw FDC being rebranded as the Family Health Team (FHT) concept. With FHT, all HCP, particularly doctors and nurses, are expected to be competent in all clinical disciplines of primary health care.

Similar concepts as FHT are seen elsewhere [7, 19] with compositions of the teams that conform to the descriptions of ‘patient care team’ as “a group of diverse clinicians who communicate with each other regularly about the care of a defined group of patients and participate in that care” [20]. While increased output is not guaranteed, team approach has been shown to be effective in task completion [19]. Other measures such as enhanced care process, patient satisfaction, continuity of care, access have also been shown to improve with the team approach [21, 22], which concurs with findings in our study.

The expansion from the lone responsible physician to an entire team was intended to better coordinate and integrate care through the cooperation of team members. Whilst elsewhere the coordination of care continuum is handled by the family physician with facilitation by electronic health records [7], in Malaysia it is handled by the Care Coordinator using manual records despite a number of the intervention clinics having electronic health records. A promising step forward in the Malaysian PHC is to have a fully integrated electronic health record system to efficiently manage patient records, hence redirect manpower to other tasks, particularly when shortages have been a constant nagging issue.

Personalised care was one of the strengths highlighted by participants in our study, which was linked with continuity of care, as well as improved quality of healthcare provided to patients. The idea of personalised physician and the team is to cultivate patients’ sense of belonging to the team [7]. This would facilitate patient empowerment and shared decision making, both of which would result in improved health outcomes. In our study, the HCP and patients expressed preference for this personalised approach as it hastened the follow-up process. However, the personalised care concept saw a small number of patients quite reluctant to see the allocated doctors due to personal preferences for other doctors. In one study, factors that influenced patients’ choice of family doctors included knowing clearly patient’s physical conditions, treatment which are timely and effective, friendliness and sincerity, one doctor keeping their medical records, and same gender as patient, amongst others [5], whereas in our study the reason given was preference towards provider personality. The matter was resolved with proper explanation by the FHT members, and acceptance to the designated team improved thereafter.

Data collection was conducted in two rounds, at early and end of intervention period. The first round assessed the immediate changes at the clinics and revealed the adaptations and modifications in terms of human resource, physical space and budget that were undertaken to implement the intervention. The second round of data collection explored the adaptiveness and acceptance to all the changes implemented, as well as the feasibility and sustainability of the intervention, which were invaluable information for future scale-up.

This study lacked in eliciting insightful feedback from the patients despite interviewers’ attempts to probe for more information. Despite being a new concept at some clinics, publicity to inform the community about the FHT initiative may have been lacking. This suggests that public engagement is crucial to ensure awareness and acceptance towards future interventions.

## Conclusion

The concept of FHT has been implemented at 20 public PHC not without expected teething problems such as manpower shortage and space constraints. Nevertheless, benefits of this initiative are clearly seen by both HCPs and patients, and appear promising in raising the potentials of achieving UHC to meet the global call. Feedbacks received in this study can inform policymakers to align future planning accordingly in order to further enhance primary health service delivery to the population. The current primary healthcare system would be prepared to scale up the FHT concept nationwide, as well as enhance its feasibility and sustainability once challenges and pertinent issues are addressed.

## Data Availability

The dataset that support the findings of this article belongs to the EnPHC study. At present, the data are not publicly available but can be obtained from the authors upon reasonable request and with the permission from the Director General of Health, Malaysia.

## Abbreviations

FDC: Family doctor concept
EnPHC: Enhanced Primary Health Care
NCD: Non-communicable disease
FHT: Family health team
HCP: Health care provider
UHC: Universal health coverage
SDG: Sustainable Development Goals
PHC: Primary health care
AMO: Assistant medical officer
IDI: In-depth interview
FGD: Focus group discussion
MOH: Ministry of Health
TOT: Training of trainers
CC: care coordinator
FMS: family medicine specialist
MCH: Maternal and child health
